# Qingxuan Zhike Granules Modulate Gut Dysbiosis and Enhance Intestinal Repair in Murine *Mycoplasma pneumonia*

**DOI:** 10.4014/jmb.2604.04045

**Published:** 2026-06-10

**Authors:** Shiyu Liu, Bowen Li, Meiling Wang, Jie Liu, Lijin Xu, Linhong Liu, Ai Liu, JingWei He, Lei Xiong, Na Wang

**Affiliations:** 1 Department of Pediatrics, Yunnan University of Traditional Chinese Medicine's First Affiliated Hospital, Kunming, Yunnan 650021, P.R. China; 2 Department of Integrative Medicine, Children’s Hospital of Fudan University, Shanghai 201102, P.R. China; 3 Department of Traditional Chinese Medicine, 905 Hospital of People's Liberation Army Navy, Shanghai 200052, P.R. China

**Keywords:** Qingxuan Zhike Granules, *Mycoplasma pneumonia*, Gut microbiota, Intestinal barrier function, Gut-lung axis

## Abstract

This study aimed to investigate the therapeutic efficacy and underlying mechanisms of Qingxuan Zhike Granules (QXZKG) in pediatric *Mycoplasma pneumoniae* pneumonia (MPP), with a specific focus on its roles in modulating gut microbiota and promoting intestinal repair via the “gut-lung axis”. A BALB/c mouse model of MPP was established for *in-vivo* experiments. Evaluations included the disease activity index (DAI), histopathological assessment of lung and intestinal tissues (H&E staining), pro-inflammatory factor levels (PCR), intestinal tight junction protein expression (Western blot), gut microbiota composition (16S rDNA sequencing), and serum lipopolysaccharide (LPS) levels. For *in-vitro* experiments, a Caco-2/RAW264.7 co-culture system was used to assess the effects of drug-containing serum on cell viability, apoptosis, inflammatory factor production, and barrier protein expression. QXZKG administration dose-dependently improved the DAI and body weight loss in MPP mice. It significantly alleviated pathological damage in both lung and intestinal tissues, reduced the expression of pro-inflammatory factors, and up-regulated the levels of intestinal tight junction proteins. Concurrently, QXZKG decreased serum LPS concentrations, restored gut microbiota diversity, and modulated its composition by increasing probiotic abundance and reducing opportunistic pathogens. *In-vitro* experiments confirmed that QXZKG-containing serum enhanced cell viability, inhibited apoptosis, reduced LPS levels, and up-regulated barrier protein expression. QXZKG is associated with modulation of the gut microbiota, enhancement of intestinal barrier function, and suppression of systemic inflammation, suggesting a potential involvement of the “gut-lung axis”. These findings provide experimental evidence for the expanded clinical application of QXZKG.

## Introduction

*Mycoplasma pneumoniae* pneumonia (MPP) is a common respiratory tract infection in children caused by *Mycoplasma pneumoniae* (MP). IIt accounts for 20%–30% of community-acquired pneumonia cases in children [1-3]. Typical clinical manifestations include fever, cough, and moist rales [4, 5]. In addition to respiratory symptoms, approximately 12% of cases progress to severe MPP, potentially accompanied by serious complications such as pulmonary necrosis or thromboembolism [6-9]. As the causative pathogen of MPP, MP can directly invade the respiratory mucosa, leading to alveolar damage, airway inflammation, and dyspnea. It can also induce extra-pulmonary manifestations via mechanisms such as the “gut–lung axis,” with intestinal dysfunction being the most prominent. These include gut microbiota imbalance, mucosal barrier disruption, and exacerbated systemic inflammatory responses, which not only worsen the patient’s condition but also increase treatment difficulty [10-12]. A major concern is that the prolonged and widespread use of macrolide antibiotics has led to a gradual global increase in macrolide-resistant MP strains, thereby reducing the efficacy of conventional anti-infective treatments. This situation necessitates urgent exploration of safe and effective alternative or adjunctive treatments [13-17].

The development and progression of respiratory disorders are greatly influenced by the gut microbiota, which plays an essential role in maintaining host homeostasis [18-21]. Under normal conditions, it interacts dynamically with the lungs via the “gut–lung axis”. This interaction takes place through a number of pathways, such as immunological balance, metabolic regulation, and barrier maintenance [22-25]. During MP infection, the diversity of gut microbiota is reduced and its structure is disrupted, with decreased abundance of probiotics (*e.g.*, *Bacteroides*, *Lactobacillus*) and overgrowth of opportunistic pathogens (*e.g.*, Proteobacteria). This microbial imbalance can disrupt intestinal barrier function through the release of endotoxins such as LPS. This may result in increased intestinal mucosal permeability, downregulation of tight junction proteins (Claudin-1, Occludin, and ZO-1), and the subsequent translocation of intestinal endotoxins into the circulation, which worsens pulmonary injury and systemic inflammation [12, 26, 27]. Therefore, restoration of gut microbiota balance and repair of intestinal barrier function have emerged as potential therapeutic targets for MPP.

In recent years, treatment research for MPP has gradually focused on gut microbiota modulation strategies, such as probiotic supplementation and fecal microbiota transplantation, which have shown certain effects in intestinal barrier repair and inflammation suppression in animal models. However, these approaches largely remain at the level of single-target exploration and have limited clinical translatability [28-31]. Traditional Chinese medicine (TCM) compound formulations, characterized by multi-component and multi-target actions, have unique advantages in the holistic regulation of infectious respiratory diseases. Previous studies have demonstrated that several TCM formulations alleviate pulmonary inflammation through the regulation of gut microbiota dysbiosis [32-35].

QXZKG is a Chinese patent medicine commonly used in pediatric respiratory tract infections. It is composed of nine medicinal ingredients: *Morus alba leaves*, *Mentha haplocalyx*, bitter apricot seed, *Platycodon grandiflorus*, *Paeonia lactiflora*, *Citrus aurantium*, dried tangerine peel, *Aster tataricus*, and licorice root. It has shown certain anti-inflammatory and tissue-protective effects in diseases such as acute lung injury [36]. However, its therapeutic efficacy and specific mechanisms against MPP remain undefined, particularly in gut microbiota regulation and intestinal barrier repair. The therapeutic effects of QXZKG in MPP may involve modulation of the gut–lung axis. We suggest that QXZKG correlates with multimodal changes in gut microbiota, barrier integrity, and inflammatory responses, which may collectively contribute to the alleviation of intestinal and pulmonary disease. Therefore, this study systematically investigated the effects of QXZKG on intestinal barrier repair and gut microbiota regulation in an MPP mouse model using both *in vitro* and *in vivo* approaches, offering scientific support for its possible therapeutic utility in the treatment of MPP.

## Experimental Methods

### *In Vivo* Grouping and Drug Administration

Six-week-old female BALB/c mice (specific pathogen-free, SPF grade) were purchased from Chengdu Dashuo Laboratory Animal Co., Ltd., (China) and housed in a controlled, pathogen-free environment under a 12-h light/dark cycle (light phase: 8:00–20:00; dark phase: 20:00–8:00) with free access to food and water. After a 14-day acclimation period, the mice were randomly divided into six groups (n = 5 per group): Control, Model, QXZKG low-dose (1 g/kg, QXZKG-L), QXZKG high-dose (5 g/kg, QXZKG-H), Bacterial depletion, and Tylosin treatment.

Mice in the Bacterial depletion group were first given drinking water containing a broad-spectrum antibiotic mixture (ampicillin 1 g/L, neomycin sulfate 1 g/L, metronidazole 1 g/L, vancomycin 0.5 g/L) for three weeks. Two days after stopping the antibiotic water, the mice received two intranasal instillations of MP (1 × 10^7^ CFU/mL, 50 μL per instillation) 12 hours apart, followed by oral gavage of QXZKG-H at a dose of 5 g/kg daily.

Mice in the Tylosin treatment group received two intranasal instillations of MP (1 × 10^7^ CFU/mL, 50 μL per instillation) 12 h apart, and 24 h later they were given oral gavage of tylosin solution at a dose of 100 mg/kg (0.4 mL per delivery) daily.

All animal experimental protocols were approved by Ethics Committee of Children’s Hospital of Fudan University [No. FELS (2025)272].

### Establishment of MPP Model

Under isoflurane anesthesia, each mouse received an intranasal instillation of 30 μL of MP bacterial suspension (1 × 10^7^ CFU/mL) once daily for three consecutive days. On day 4, blood samples were collected from the retro-orbital venous plexus, and MP DNA was extracted and detected following the manufacturer’s protocol with a Mycoplasma nucleic acid detection kit. A positive result was considered indicative of successful model establishment.

### Preparation of Drug-Containing Serum and *In Vitro* Grouping

Four groups of BALB/c mice were randomly assigned: a positive drug group, a low-dose group receiving Qingxuan Zhike Granules (QXZKG-L), a high-dose group receiving Qingxuan Zhike Granules (QXZKG-H), and a normal control group. For seven consecutive days, the normal control group was administered an equivalent volume of physiological saline by gavage once daily. Blood samples were taken from the retro-orbital venous plexus one hour following the final injection. Following centrifugation, the serum was sterilized through membrane filtration, inactivated for 30 min at 56°C in a water bath, and stored at -20°C until use until it was needed. A co-culture system including alveolar macrophage RAW264.7 cells and intestinal epithelial Caco-2 cells was then established. The experimental groups included a normal control group, an MP model group (cells infected with MP at a concentration of 1 × 10^7^ CCU/mL for 24 h), QXZKG-L and QXZKG-H groups, and a positive drug group (azithromycin) were all included in the experimental setup.

### Disease Activity Index (DAI)

Each mouse’s body weight, food and water intake, activity level, mental state (including specific behaviors like shivering, hunching, piloerection, and irregular breathing), and stool characteristics were recorded daily throughout the MP infection and treatment period. The mean of the ratings given to the three indicators—body weight change, mental state, and stool condition—was used to calculate the clinical scores. Table 1 lists the clinical assessment criteria used in this investigation.

### Hematoxylin–Eosin (HE) Staining

To assess pathological damage in the tissues of the lung and colon, HE staining was used. Colon and lung tissues were removed from the mice after they were put to sleep and washed with PBS. These tissues were embedded in paraffin wax, fixed for 48 h in a 4% paraformaldehyde solution, and then cut into sections that were 5 μm thick. An HE staining kit was used to stain the sections after they had been deparaffinized in xylene and rehydrated using a gradient ethanol series. In order to evaluate histological damage, the sections were finally mounted with neutral balsam and examined under a light microscope.

### Immunohistochemistry (IHC)

Sections of colon paraffin made as previously mentioned were incubated for two hours at 65°C. The sections were then cleaned with running water, rehydrated using a sequence of graded ethanol, and deparaffinized using xylene. The antigen retrieval process was performed in a pH 6.0 sodium citrate buffer. The UltraSensitive^TM^ SP (mouse/rabbit) IHC kit was then used to prevent non-specific binding and endogenous peroxidase activity. Following an overnight incubation at 4? with primary antibodies that target ZO-1, Claudin-1, and Occludin, the sections were treated for 10 minutes at 37°C with a biotin-labeled secondary antibody (anti-rabbit/anti-mouse IgG), and then for a further 10 minutes with streptavidin–peroxidase. The sections were then counterstained with Mayer's haematoxylin solution after being developed for five minutes at room temperature using 3,3’-diaminobenzidine (DAB) and carefully washed under running water. Following DAB development, the staining results were inspected under a microscope in order to evaluate the results of IHC.

### 16S rDNA High-Throughput Sequencing

Colonic content samples were collected into sterile centrifuge tubes. Total DNA was extracted using the CTAB method. DNA quantity and quality were assessed, and the V3–V4 region of the 16S rRNA gene was amplified using universal primers. The amplified products were purified and quantified before sequencing. Paired-end sequencing was performed on an Illumina MiSeq platform with a read length of 300 bp. The average sequencing depth was approximately 50,000 reads per sample. After demultiplexing, adapter and barcode sequences were removed, and paired-end reads were merged and quality-filtered using DADA2 with the following parameters: truncation length set to 240 bp for forward reads and 200 bp for reverse reads, and a maximum expected error of 2. Taxonomy was assigned using the naive Bayesian classifier against the SILVA database (version 138). Alpha diversity (Shannon and Simpson indices) and beta diversity (weighted and unweighted UniFrac, principal coordinate analysis) were calculated. All downstream statistical analyses were performed using R packages (vegan, phyloseq).

### Caco-2/RAW264.7 Co-Culture System

Caco-2 and RAW264.7 cells were co-cultured in Transwell inserts (0.4 μm pore size), with Caco-2 cells on the apical side and RAW264.7 cells in the basolateral compartment. Caco-2 cells were differentiated for 21 days to form tight junctions. The co-culture was exposed to MP (1 × 10^7^ CCU/mL) and QXZKG-containing serum for 24 h. Caco-2 cells were used at passages 20–30 and RAW264.7 cells at passages 5–15.

### Western Blotting (WB)

Western blotting (WB) was used to assess the protein levels in each experimental group. Initially, proteins were extracted from cells or tissues on ice, and a BCA protein assay kit was employed to determine protein concentrations. The samples were then diluted to a uniform 30 μg. Proteins were separated by 12% SDS–polyacrylamide gel electrophoresis (SDS-PAGE), and the transfer duration was adjusted based on the molecular weight of the target proteins. Following electrophoresis, the membranes underwent blocking for 2 h at room temperature with 5% non-fat milk, and were then incubated with primary antibodies overnight at 4°C. The membranes were then treated with secondary antibodies coupled to horseradish peroxidase (HRP) for two hours. Lastly, protein bands were visualized using chemiluminescence imaging equipment.

### RNA Extraction and Quantitative PCR (qPCR)

Gene expression changes were confirmed at the transcriptional level using qPCR. We extracted total RNA from colon tissues or cells using the RNeasy Plus kit. Reverse transcription was performed using the BioRT Master HisSensi cDNA First-Strand Synthesis Kit following the manufacturer’s protocol. qPCR was conducted using a 2720 thermal cycler and an ABI 7500 PCR system with SYBR Green I as the fluorescent dye. Table 2 presents the primers used for PCR amplification.

### Data Analysis

Values are shown as mean ± standard deviation (SD). Differences between two groups were tested using a two-tailed Student’s t-test, while tests between multiple groups were performed using analysis of variance (ANOVA). A *p*-value less than 0.05 was considered statistically significant. The significance levels were designated as follows: * *p* <0.05, ** *p* <0.01, and *** *p* <0.001.

## Experimental Results

### QXZKG Improves Overall Disease Activity in Mice with MP Pneumonia

**Fig. 1:**
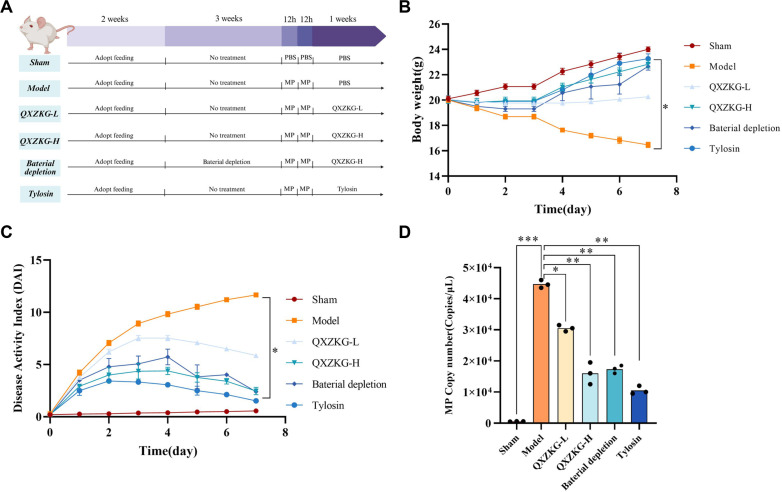
QXZKG improves overall disease activity in mice with MP pneumonia. (**A**) Animal study experimental design, generated with BioRender and published with authorization. (**B**) Body weight changes in mice. (**C**) Pneumonia DAI scores of each group. (**D**) MP loads in each group. *p < 0.05, **p < 0.01, **p < 0.001.

This section was designed to evaluate the regulatory effects of QXZKG on the overall health status of MP-infected mice. The results showed that control mice maintained stable body weight, had low DAI scores (covering body weight, food and water intake, activity level, and stool characteristics), and exhibited extremely low MP loads in lung tissues (Fig. 1A–1D), indicating good physiological status. In the model group, MP infection resulted in significant body weight loss, a marked increase in DAI scores, and a sharp rise in MP load in lung tissues, suggesting severe health deterioration. QXZKG intervention improved these indicators in a dose-dependent manner. The QXZKG-L group showed moderate improvement, whereas the QXZKG-H group demonstrated superior efficacy: body weight was restored to a level close to that of the control group, DAI scores were markedly reduced compared with both the model and QXZKG-L groups, and MP load reduction exceeded that in the bacterial depletion group. Although slightly less effective than the tylosin group, high-dose QXZKG effectively suppressed overall disease activity induced by MP infection and mitigated pathogen-induced damage, highlighting its therapeutic potential in improving the general condition of infected mice.

### QXZKG Attenuates Pathological Damage in Lung and Colon Tissues of Mice with MP Pneumonia

**Fig. 2:**
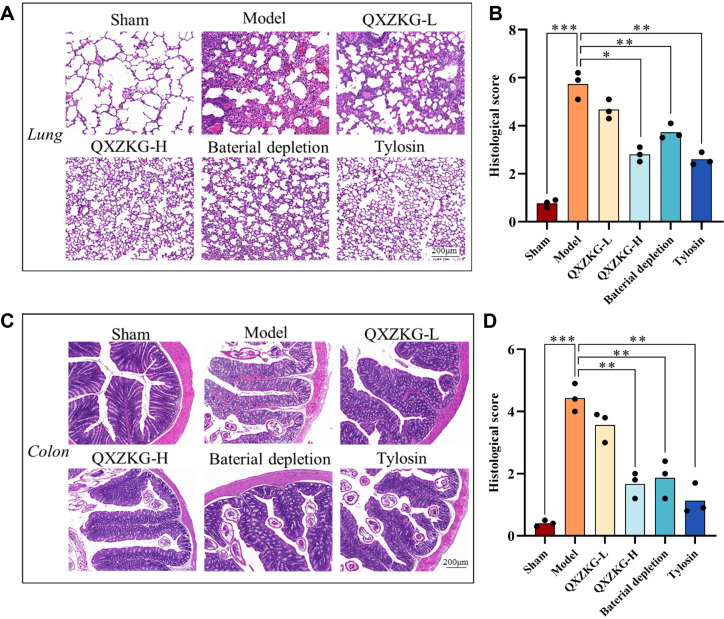
QXZKG attenuates pathological damage in lung and colon tissues of mice with MP pneumonia. (**A**) HE staining of intestinal tissue samples from each group. (**B**) Histopathological scores of intestinal tissues for each group. (**C**) HE staining of lung tissue samples from each group. (**D**) Histopathological scores of lung tissues for each group. *p < 0.05, **p < 0.01, ***p < 0.001.

Histopathological changes were assessed through HE staining. In the control group, the lung and colon tissues displayed morphologically intact structures, with no notable pathological alterations. In the model group, lung tissues showed alveolar structure destruction, hemorrhage, congestion, thickened alveolar walls, and extensive neutrophil infiltration (Fig. 2A and 2B). Colon tissues showed significant goblet cell loss, disruption of crypt architecture, damage to the mucosal layer, and prominent inflammatory cell infiltration (Fig. 2C and 2D)—findings that are indicative of pathological damage induced by MP infection. After intervention with QXZKG, pathological injury was mitigated in the QXZKG-L group,while the QXZKG-H group showed more substantial histopathological improvement. In the latter, alveolar structures were largely restored, hemorrhage and inflammatory infiltration were markedly reduced, and histological scores were considerably lower compared with both the model and QXZKG-L groups. In the colon, goblet cell loss was reduced, crypt architecture and mucosal integrity were improved, and inflammatory infiltration was diminished. These results indicate that high-dose QXZKG effectively attenuates MP-induced tissue injury in a dose-dependent manner.

### QXZKG Inhibits the Expression of Inflammatory Cytokines in the Lungs and Intestines of Mice with MP Pneumonia

**Fig. 3:**
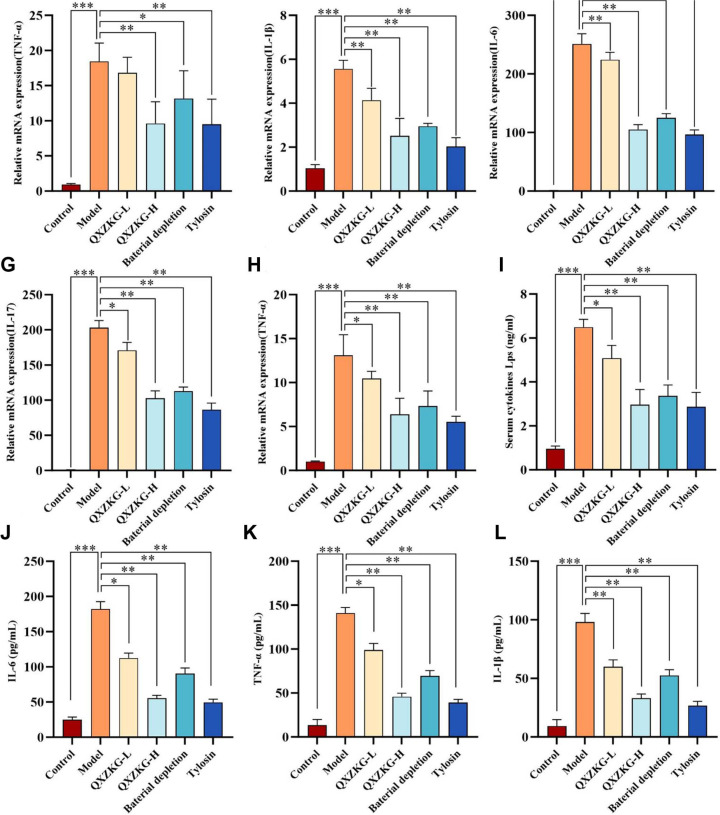
QXZKG suppresses the expression of inflammatory cytokines in the lungs and intestines of mice with MP pneumonia. (**A–D**) Levels of IL-1β, IL-6, IL-17, and TNF-α in intestinal tissues. (**E–H**) Levels of IL-1β, IL-6, IL-17, and TNF-α in lung tissues. (**I**) Serum LPS concentrations (measured by ELISA). (**J**) Serum IL-6 levels. (**K**) Serum TNF-α levels. (**L**) Serum IL-1β levels. *p < 0.05, **p < 0.01, ***p < 0.001.

To investigate QXZKG's anti-inflammatory mechanism, mRNA expression of pro-inflammatory cytokines (IL-17, IL-6, IL-1β, TNF-α) was detected in lung and intestinal tissues by qPCR. Compared with the control group, the model group showed significantly elevated cytokine expression, while QXZKG treatment reduced it in a dose-dependent manner, with the high-dose group approaching the effect of tylosin (Fig. 3A-3H). Serum LPS levels, which were elevated in the model group, were significantly lowered by high-dose QXZKG (Fig. 3I). ELISA further revealed that serum levels of IL-6, TNF-α, and IL-1β were markedly increased in the model group and dose-dependently reduced by QXZKG (Fig. 3J-3L). These results suggest that QXZKG exerts systemic anti-inflammatory effects.

### QXZKG Enhances the Integrity of the Intestinal Barrier in Mice with MP Pneumonia

**Fig. 4:**
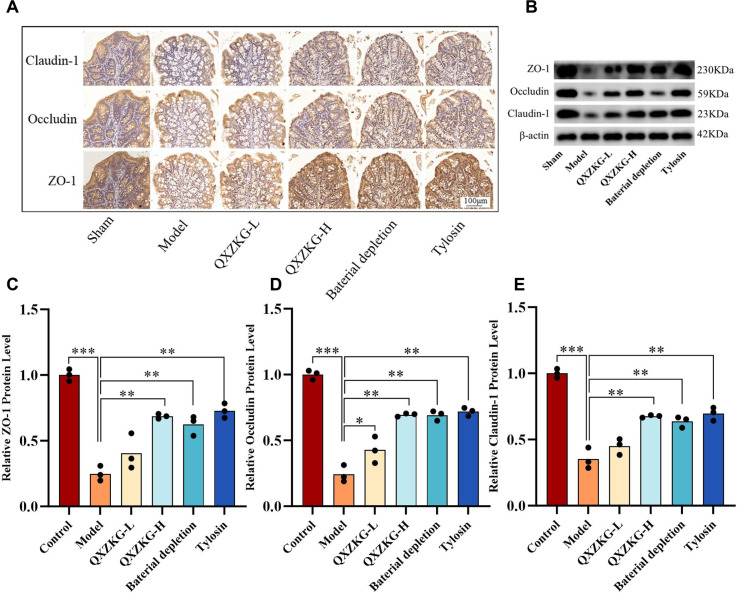
QXZKG enhances intestinal barrier integrity in mice with MP pneumonia. (**A**) Immunohistochemical detection of Claudin-1, Occludin, and ZO-1 in colon tissue samples. (B-E) Western blot detection of Claudin-1, Occludin, and ZO-1 protein levels in colon tissues. *p < 0.05, **p < 0.01, ***p < 0.001.

Impaired intestinal barrier function is a major extrapulmonary symptom of MP infection [37]. In this section, changes in the expression of tight junction proteins were assessed using IHC and WB to determine QXZKG’s protective effects on the intestinal barrier. In the control group, Claudin-1, Occludin, and ZO-1 were highly and consistently expressed in colon tissues (Fig. 4A). In the model group, MP infection resulted in a significant decrease in positive staining intensity of these proteins, indicating structural damage of the intestinal barrier. After QXZKG therapy, the low-dose group’s positive staining intensity was partially restored, whereas the high-dose group’s recovery was more dramatic, outperforming the bacterial depletion group. The high-dose QXZKG group showed weaker staining intensity than the tylosin group but significantly stronger staining than the model group, yet still showed a notable increase relative to the model group. WB data supported these findings: Claudin-1, Occludin, and ZO-1 protein levels were high in the control group (Fig. 4B-E); in the model group, athe expression levels of all three proteins were significantly reduced. Following QXZKG treatment, protein levels rose in a dose-dependent manner, with the high-dose group exhibiting greater upregulation than the low-dose and bacterial depletion groups. Although its efficacy was slightly lower than that of tylosin, the differences from the model group were statistically significant.

### QXZKG Modulates the Structure and Composition of the Gut Microbiota in Mice with MP Pneumonia

**Fig. 5:**
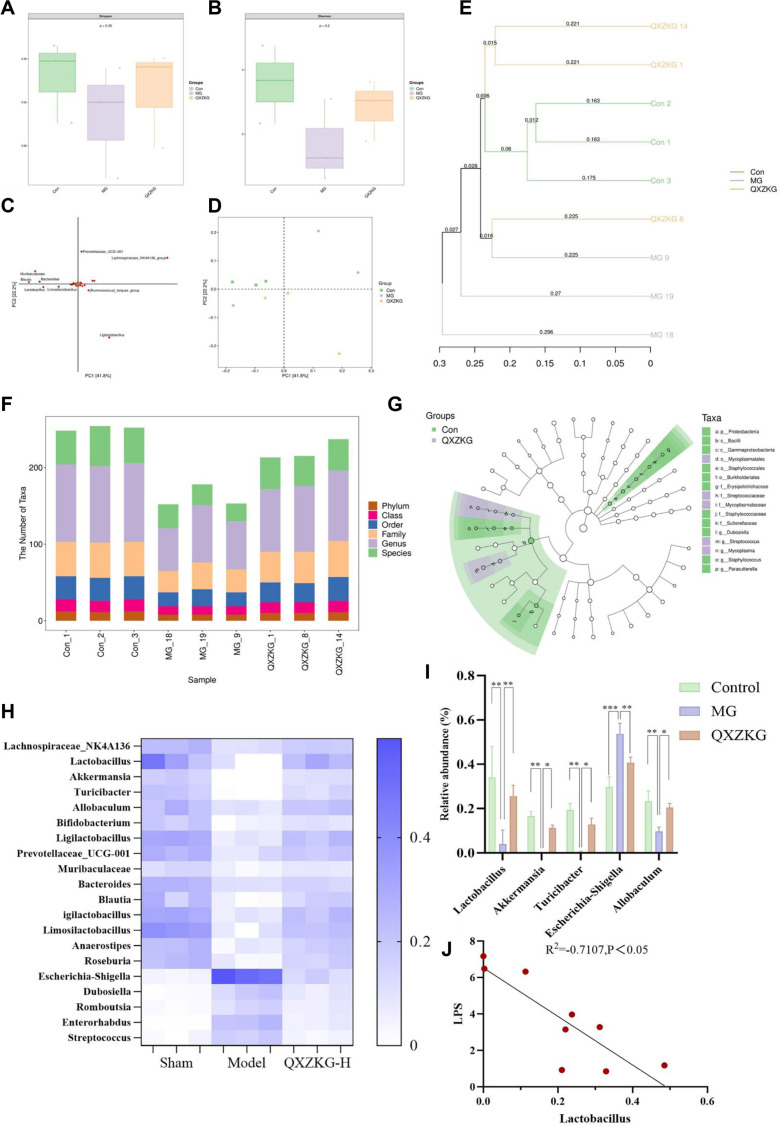
QXZKG modulates the structure and composition of gut microbiota in mice with MP pneumonia. (**A**) Simpson index and (**B**) Shannon index, both for alpha diversity analysis. (**C–D**) Principal coordinate analysis (PCoA). (**E**) Sample clustering analysis. Data are presented as mean ± standard deviation. (**F**) Taxonomic composition of gut microbiota in each group at the phylum, class, order, family, genus, and species levels. (**G**) LEfSe analysis of bacterial taxa with significantly different relative abundance among groups. (**H**) Relative abundance of gut microbiota at the genus level in colonic contents. (**I**) Relative abundance of selected bacterial taxa with known functional relevance: *Lactobacillus* (commonly associated with beneficial effects) and Proteobacteria (a phylum containing LPS-producing opportunistic pathogens). (**J**) Correlation between *Lactobacillus* and serum LPS (r = -0.7107, *p* < 0.05). **p < 0.01.

16S rDNA sequencing revealed that MP infection reduced gut microbiota diversity, as shown by lower Shannon and Simpson indices in the model group compared with the control group, while high-dose QXZKG restored diversity to control levels (Fig. 5A and 5B). PCoA and clustering analyses (Fig. 5C-5E) demonstrated that the microbial community of the QXZKG group resembled that of the control group, distinct from the model group. MP infection altered the overall composition and richness of the gut microbiota, which was corrected by QXZKG (Fig. 5F). LEfSe analysis identified differentially abundant taxa across groups (Fig. 5G). At the genus level (Fig. 5H), MP infection reduced *Lactobacillus* and increased Proteobacteria, and QXZKG reversed these changes. The relative abundance of selected taxa is shown in Fig. 5I. Importantly, *Lactobacillus* abundance was negatively correlated with serum LPS levels (r = -0.7107, *p* < 0.05, Fig. 5J).

### *In Vitro* Effects of QXZKG-Containing Serum on MP-Related Cell Models

**Fig. 6:**
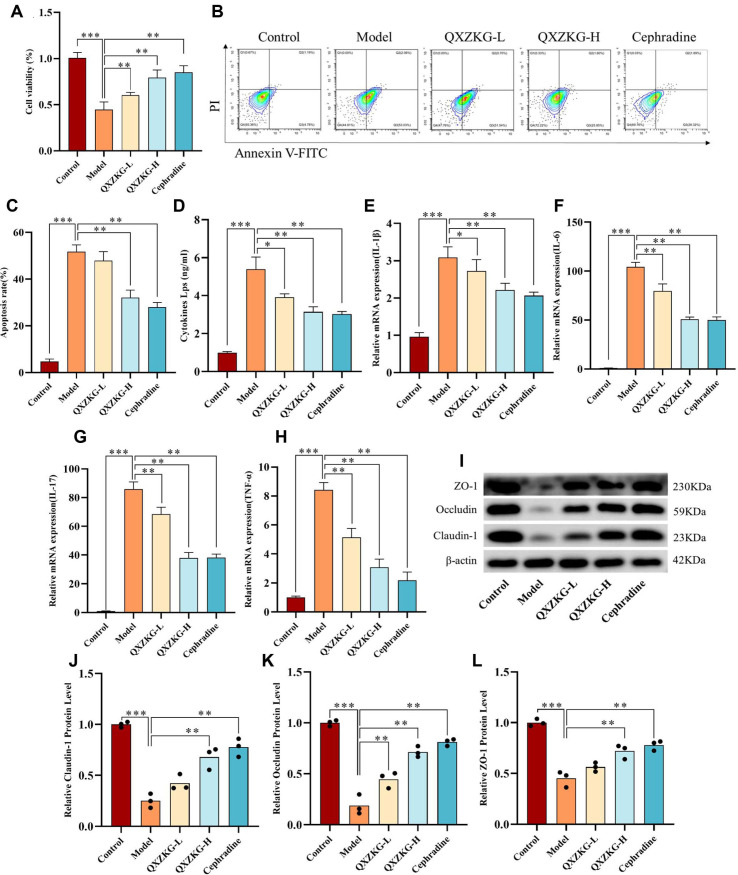
*In vitro* effects of QXZKG-containing serum on MP-related cell models. (**A**) Cell viability assessed by Cell Counting Kit-8 (CCK-8). (**B–C**) Flow cytometric detection of apoptosis. (**D**) LPS levels in cell lysates measured by ELISA. (**E–H**) Levels of IL-1β, IL-6, IL-17, and TNF-α in cell lysates. (**I–L**) Western blot detection of Claudin-1, Occludin, and ZO-1 protein levels in cell lysates. **p < 0.01.

To confirm the effects of QXZKG, *in vitro* tests were carried out using a Caco-2/RAW264.7 co-culture system to assess the impact of QXZKG-containing serum. The CCK-8 assay findings showed that cell viability was considerably reduced in the model group, whereas high-dose QXZKG serum dramatically enhanced cell viability (Fig. 6A), indicating a protective effect against cellular injury. Flow cytometry analysis revealed a significant increase in the model group's apoptosis rate, whereas high-dose QXZKG-containing serum reduced apoptosis in a dose-dependent manner (Fig. 6B and 6C), which was consistent with changes in LPS levels in cell lysates determined via ELISA (Fig. 6D). PCR results showed that QXZKG-containing serum reduced the expression of pro-inflammatory cytokines, including IL-17 and IL-6, in cells (Fig. 6E-6H), while Western blot analysis confirmed that QXZKG enhanced the protein levels of Claudin-1, Occludin, and ZO-1 in Caco-2 cells (Fig. 6I-6L). The results from *in vitro* experiments were consistent with the in vivo data, demonstrating that QXZKG can preserve the intestinal barrier, inhibit inflammatory responses, and reduce apoptosis, thereby intervening in MP infection.

## Discussion

MPP involves not only pulmonary inflammation but also gut microbiota dysbiosis and intestinal barrier disruption. The limitations of macrolide antibiotics, including resistance and gut microbial imbalance, highlight the need for multi-targeted therapies [10, 38, 39]. QXZKG, a traditional Chinese medicine formula for pediatric respiratory diseases, was therefore investigated for its potential to act through the gut–lung axis.

Our *in vivo* data showed that MP infection induced systemic changes: weight loss, elevated DAI scores, lung and colon damage, increased pro-inflammatory cytokines (IL-17, IL-6, IL-1β, TNF-α) in both tissues, reduced tight junction proteins (Claudin-1, Occludin, ZO-1), and raised serum LPS. QXZKG dose-dependently reversed these abnormalities, and the *in vitro* co-culture system confirmed that QXZKG-containing serum directly enhanced intestinal epithelial barrier function and reduced inflammation. These results suggest a potential involvement of the gut–lung axis in the effects of QXZKG, though causal evidence remains to be established.

MP infection led to a significant decrease in gut microbiota diversity in mice, with a marked reduction in the abundance of Bacteroidetes (a phylum containing many commensal bacteria that contribute to gut homeostasis) and a massive proliferation of Proteobacteria (a phylum that includes opportunistic pathogens such as *Escherichia coli*), thereby disrupting intestinal homeostasis. QXZKG treatment was associated with a shift in microbiota composition: enrichment of taxa that are generally considered beneficial (*e.g.*, *Lactobacillus*) and suppression of potentially harmful taxa (*e.g.*, Proteobacteria). This effect may be related to microbiota-derived metabolites, which, through systemic circulation, suppress neutrophil infiltration in the lungs, restore intestinal immune homeostasis, and reduce the transmission of pro-inflammatory signals to the lungs. For example, our data revealed a significant negative correlation between *Lactobacillus* abundance and serum LPS levels (r = -0.7107, *p* < 0.05, Fig. 5J). *Lactobacillus* species are known to produce short-chain fatty acids (SCFAs) such as butyrate, which can upregulate tight junction proteins (Claudin-1, Occludin, ZO-1) and reduce intestinal permeability, thereby limiting LPS translocation into the circulation. Conversely, the expansion of Proteobacteria (*e.g.*, *E. coli*), a major source of endotoxin, directly contributes to elevated serum LPS. QXZKG treatment concurrently increased probiotic *Lactobacillus* and reduced opportunistic Proteobacteria, providing a plausible mechanistic link between microbiota remodeling, reduced endotoxemia, and enhanced intestinal barrier function. Compared with the conventional antibiotic tylosin, which may exacerbate secondary dysbiosis of the gut microbiota, QXZKG, through promoting beneficial bacteria while suppressing pathogenic bacteria, may better align with the pathological repair of MPP, which may better support the restoration of gut–lung axis homeostasis in MPP. Furthermore, MP infection reduced the expression of tight junction proteins, impaired the mechanical barrier of the intestinal mucosa, and aggravated LPS translocation as well as systemic inflammatory responses. QXZKG dose-dependently increased the expression of tight junction proteins, thereby decreasing endotoxin entry into the bloodstream and attenuating pulmonary inflammation. These findings suggest a potential synergistic relationship between intestinal barrier repair and microbiota: a healthy microbiota can promote tight junction protein synthesis via its metabolites, while an intact intestinal barrier provides a favorable environment for the colonization of beneficial bacteria.

To further assess whether the protective effects of QXZKG depend on an intact gut microbiota, we included a Bacterial Depletion group in which mice were pretreated with broad-spectrum antibiotics to deplete gut bacteria prior to MP infection and QXZKG-H treatment. Our results showed that antibiotic-induced microbiota depletion significantly attenuated the beneficial effects of QXZKG. Compared with the QXZKG-H group, the Bacterial Depletion group exhibited lower microbial diversity, reduced expression of intestinal tight junction proteins (Claudin-1, Occludin, ZO-1), elevated serum LPS levels, and more severe pulmonary histopathology, although these parameters remained better than those in the Model group. These findings indicate that the therapeutic effects of QXZKG are partially mediated by the gut microbiota. However, the incomplete abrogation of efficacy also suggests that QXZKG may exert additional microbiota-independent actions (*e.g.*, direct anti-inflammatory or epithelial barrier-protective effects), which warrant further investigation.

Compared with tylosin, the overall improvement of MPP by QXZKG was slightly weaker. Tylosin showed superior efficacy in rapidly reducing MP load, reversing body weight loss, and alleviating lung histopathological damage, which is expected given its direct antibacterial activity. However, both QXZKG and tylosin effectively restored intestinal barrier integrity, as evidenced by increased expression of tight junction proteins. Notably, QXZKG exhibited a distinct advantage in gut microbiota modulation: it increased the abundance of beneficial bacteria such as *Lactobacillus* and reduced opportunistic pathogens such as Proteobacteria, whereas tylosin, despite its strong anti-infective effect, may be associated with a broader impact on gut microbial diversity. Additionally, the potential cytotoxicity of high-dose tylosin cannot be overlooked, while QXZKG showed a favorable safety profile. Therefore, rather than one agent being superior, our data support a complementary role for QXZKG as an adjunct to tylosin, particularly in mild to moderate or macrolide-resistant MPP, where it may help preserve gut microbiota homeostasis and reduce antibiotic dosage. These findings suggest a potential adjunctive strategy for long-term management of MPP through modulation of the gut–lung axis.

This study still has several limitations. First, the specific active monomer components in QXZKG responsible for its core effects remain unidentified, and a detailed chemical profile (*e.g.*, HPLC or LC-MS fingerprinting) of the batch used has not been established, leaving the precise molecular targets and future reproducibility to be addressed. Second, the specific signaling pathways of the “gut–lung axis” (*e.g.*, TLR4/NF-κB, MAPK) have not been deeply explored. Third, direct mechanistic validation—such as microbiota depletion, fecal microbiota transplantation, or pathway inhibition—has not been performed in this study; therefore, causal evidence linking gut microbiota changes to pulmonary outcomes along the gut–lung axis remains to be established in future investigations. Fourth, clinical translational evidence is lacking, as the relationship between gut microbiota characteristics in pediatric MPP patients and QXZKG efficacy has not been analyzed. Fifth, the sample sizes used in this study (n = 5 per group for 16S rRNA sequencing and most biochemical assays, and n = 3 per group for Western blot) are relatively modest; although these are consistent with common practice in the field, independent replication with larger cohorts would further strengthen the conclusions. Sixth, tylosin is a veterinary macrolide, whereas azithromycin or doxycycline are the standard clinical options for pediatric MPP; therefore, the translational implications of our comparison should be interpreted with caution, and future studies using clinically relevant antibiotics are warranted.

## Conclusion

In summary, QXZKG was associated with therapeutic effects in MPP model mice, and these effects correlated with changes that may involve the “gut–lung axis”: on the one hand, increased abundance of beneficial bacteria, decreased opportunistic pathogen levels, and restored microbial diversity; on the other hand, dose-dependent upregulation of tight junction proteins (Claudin-1, Occludin, ZO-1), strengthened the intestinal barrier, and reduced LPS levels. These effects were more pronounced in the high-dose group, which also further reduced the overexpression of pro-inflammatory cytokines (including IL-17 and IL-6) in lung tissues, alleviated pathological changes such as congestion, inflammatory infiltration, and alveolar damage, and improved colonic mucosal injury. These findings provide a reference for understanding how multi-component TCM formulations may modulate gut–lung axis-associated diseases through multiple pathways., QXZKG provides preclinical experimental evidence suggesting a potential role of the “gut–lung axis” in MPP treatment, warranting further investigation in future translational studies.

## Figures and Tables

**Table 1:** Calculated DAI score.

Score	Weight loss	Mental state	Stool character
0	0	Normal	Normal formed
1	0.1–2.5%	Curling up	
2	2.6–5.0%	Curling up, shivering, eating less	Loosen stool
3	5.1–7.5%	Curling up, shivering, eatingless, pilo-erection	
4	7.6–10%	Curling up, shivering, eating less, pilo-erection paralysis ofhind limbs, abnormal breathing	Diarrhea

**Table 2:** Primer sequence used for mRNA RT-qPCR.

Gene	Sequences (5′–3′)
IL-17	F: ACTACCTCAACCGTTCCACG R: TTCCCTCCGCATTGACACAG
IL-1β	F: ATCAACCAACAAGTGATATTCTCCAT R: GGGTGTGCCGTCTTTCATTAC
IL-6	F: GAGGATACCACTCCCAACAGACC R: AAGTGCATCATCGTTGTTCATACA
TNF-α	F: AACCTCCTCTCTGCCGTCAA R: AAGTAGACCTGCCCGGACTC
β-actin	F: GTCAGGTCATCACTATCGGCAAT R: AGAGGTCTTTACGGATGTCAACGT
